# Unconjugated Bilirubin Exposure Impairs Hippocampal Long-Term Synaptic Plasticity

**DOI:** 10.1371/journal.pone.0005876

**Published:** 2009-06-11

**Authors:** Fang-Yu Chang, Cheng-Che Lee, Chiung-Chun Huang, Kuei-Sen Hsu

**Affiliations:** 1 Department of Pharmacology, College of Medicine, National Cheng Kung University, Tainan, Taiwan; 2 Center for Gene Regulation and Signal Transduction Research, National Cheng Kung University, Tainan, Taiwan; The Research Center of Neurobiology - Neurophysiology of Marseille, France

## Abstract

**Background:**

Jaundice is one of the most common problems encountered in newborn infants, due to immaturity of hepatic conjugation and transport processes for bilirubin. Although the majority of neonatal jaundice is benign, some neonates with severe hyperbilirubinemia develop bilirubin encephalopathy or kernicterus. Accumulation of unconjugated bilirubin (UCB) in selected brain regions may result in temporary or permanent impairments of auditory, motor, or cognitive function; however, the molecular mechanisms by which UCB elicits such neurotoxicity are still poorly understood. The present study is undertaken to investigate whether prolonged exposure of rat organotypic hippocampal slice cultures to UCB alters the induction of long-term synaptic plasticity.

**Methodology/Principal Findings:**

Using electrophysiological recording techniques, we find that exposure of hippocampal slice cultures to clinically relevant concentrations of UCB for 24 or 48 h results in an impairment of CA1 long-term potentiation (LTP) and long-term depression (LTD) induction in a time- and concentration-dependent manner. Hippocampal slice cultures stimulated with UCB show no changes in the secretion profiles of the pro-inflammatory cytokines, interleukin-1β and tumor necrosis factor-α, or the propidium ioide uptake. UCB treatment produced a significant decrease in the levels of NR1, NR2A and NR2B subunits of *N*-methyl-D-aspartate (NMDA) receptors through a calpain-mediated proteolytic cleavage mechanism. Pretreatment of the hippocampal slice cultures with NMDA receptor antagonist or calpain inhibitors effectively prevented the UCB-induced impairment of LTP and LTD.

**Conclusion/Significance:**

Our results indicate that the proteolytic cleavage of NMDA receptor subunits by calpain may play a critical role in mediating the UCB-induced impairment of long-term synaptic plasticity in the hippocampus. These observations provide new insights into the molecular mechanisms underlying UCB-induced impairment of hippocampal synaptic plasticity which, in turn, might provide opportunities for the development of novel therapeutic strategies that targets these pathways for treatment.

## Introduction

Bilirubin, an oxidative end product of heme catabolism, is excreted by liver after glucuronidation by hepatic uridine diphosphate-glucuronyl transferase [1]. Hyperbilirubinemia is one of the most common clinical phenomena observed in the neonatal period. Nearly all newborn infants may experience temporary, mild to moderate ‘physiological’ jaundice, due to immaturity of hepatic conjugation and clearance processes for unconjugated bilirubin (UCB) [2]. In the vast majority of cases, neonatal jaundice represents a benign phenomenon and the modest elevation of plasma UCB may exert neuroprotective effects owing to its antioxidant properties [3]. Some newborns, however, especially preterm infants with hemolytic diseases, the concentration of UCB may rise to higher levels that cause bilirubin encephalopathy or may progress to kernicterus resulting in severe neurological dysfunctions [2]. The brain regions particularly vulnerable to UCB toxicity include the cerebellum, cochlear and oculomotor nuclei of the brain stem, the hippocampus, and the basal ganglia [4]. The core clinical features of UCB encephalopathy may range from mild mental retardation and subtle cognitive disturbances to deafness and severe cerebral palsy, seizure or death from kernicterus [2], [5]. Data from several prospective controlled studies have revealed cognitive disturbances in children with elevated levels of UCB in the infant period [6]–[9]. Hence, considerable interest is now focused on understanding the molecular mechanisms by which UCB exerts such neurodevelopmental abnormality in order to generate effective therapeutic strategies targeting these pathways for treatment. Because mechanistic studies in humans are limited, a plausible way to address this question is to use an experimental model that simulates the clinically relevant UCB concentration exposure in the developing brain.

Activity-dependent persistent synaptic modifications are generally thought to be the cellular mechanisms underlying the refinement of neuronal connections in the developing nervous systems [10], [11] and contributing to the processes of learning and memory in the mature brain [12], [13]. Persistent synaptic modifications can involve alterations in both of the function of synaptic transmission and the structure of neuronal connections. Studies of synaptic plasticity have shown that repetitive electrical activity can rapidly induce persistent changes in the strength of synaptic transmission, known as long-term potentiation (LTP) and long-term depression (LTD) [14], [15]. The molecular mechanisms of LTP and LTD have been extensively characterized [16], especially in hippocampus, has been implicated in memory formation of spatial learning tasks in rodents an area implicated in spatial memory formation in rodents [17]. Induction of LTP and LTD in the CA1 region of the hippocampus involves numerous protein kinases and/or phosphatases [13], [18], which are believed to be critical for the translation of electrical activity into persistent subcellular alterations that may modulate synaptic strength. Interestingly, the adult rats received a bolus intravenous injection of either 30 mg/kg or 60 mg/kg of bilirubin has been found to inhibit the induction of LTP in the hippocampal CA3 region in vivo [19]. Thus, we assumed that UCB might affect the cognitive development during neonatal life through changes in the bidirectional hippocampal synaptic plasticity.

In the present study, we used rat organotypic hippocampal slice cultures, which maintain cytoarchitecture of the intact brain and are well suited for prolonged pharmacological treatments [20], [21], to investigate whether prolonged clinically relevant UCB concentration treatment may alter the induction of CA1 LTP and LTD and characterize the possible underlying mechanisms. Our data constitute the first evidence that UCB-induced impairment of CA1 LTP and LTD induction in the hippocampus occurs through the stimulation of calpain-mediated proteolytic cleavage of N-methyl-D-aspartate (NMDA) receptor subunits.

## Results

### Effect of prolonged UCB exposure on basal synaptic transmission

**Figure 1:**
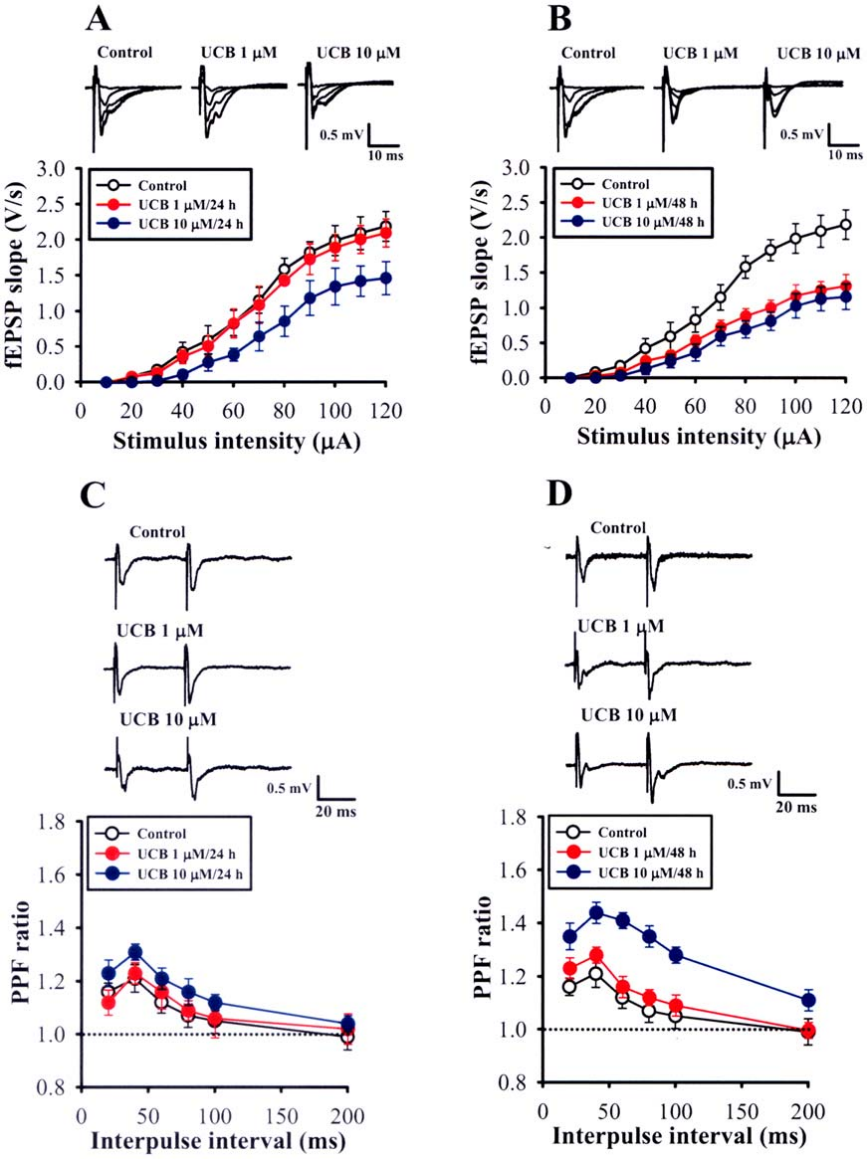
Effects of prolonged UCB exposure on basal synaptic transmission and paired-pulse facilitation (PPF). (A, B) Input-output curves of field excitatory postsynaptic potentials (fEPSPs; V/s) versus stimulus intensity (µA) at the Schaffer collateral-CA1 synapses of hippocampal slice cultures in the absence (control) or presence of 1 or 10 µM UCB for 24 h (A) or 48 h (B). Inset shows example fEPSPs (average of three responses) recorded in slices from control and UCB-treated slices. (C, D) Comparison of PPF ratio in slices from control and treated with 1 or 10 µM UCB for 24 h (C) and 48 h (D). The plot summarizes facilitation of the second fEPSP slope relative to first one as a function of the interpulse intervals of 20 to 200 ms. Inset shows example PPF (average of three responses) obtained with interpulse interval of 40 ms in slice from control and UCB-treated slices. Error bars indicate SEM.

We initially examined whether the basal synaptic transmission at the Schaffer collateral-CA1 synapses was altered by prolonged UCB exposure. The stimulus-response relationships for extracellular field excitatory postsynaptic potentials (fEPSPs) obtained from UCB exposure and control slices were compared. As shown in Figure 1A, exposure of UCB at a concentration of 1 µM for 24 h had no effect on the stimulus-response curve, maximal response, and fEPSP waveform. In contrast, when exposure of slices to 1 µM UCB for 48 h or the concentration of UCB was increased to 10 µM for 24 or 48 h, the stimulus-response curve significantly exhibited a rightward shift (*p*<0.05) and the maximal response of fEPSPs was significantly reduced (Figure 1A and B). In order to test whether the deficit in fEPSPs were due to the alterations in the excitability of the afferent fibers, we measured the presynaptic fiber volley amplitude in the presence of AMPA/kainate receptor antagonist 6-cyano-7-nitroquinoxaline-2,3-dione (CNQX, 20 µM) and NMDA receptor antagonist D-2-amino-5-phosphonovalerate (D-APV, 50 µM) and found no difference in slices treated 10 µM UCB for 24 or 48 h compared with the control slices at all stimulus intensities measured (Figure S1). These results indicate that prolonged treatment with a higher concentration of UCB (10 µM) can produce a decrease in basal synaptic transmission without altering the excitability of afferent fibers. Given the similarity of the results with 24 and 48 h control slices, the two sets of results were averaged throughout the work.

To determine whether prolonged UCB exposure alters the presynaptic function, we examined the paired-pulse facilitation (PPF), a transient form of presynaptic plasticity in which the second of two closely spaced stimuli elicits enhanced transmitter release [22]. As shown in Figure 1C, pairs of presynaptic fiber stimulation pulses delivered at interpulse intervals of 20, 40, 60, 80, and 100 ms evoked nearly identical amounts of PPF in slices from UCB (1 µM or 10 µM) exposure for 24 h and control. In contrast, exposure of slices with 10 µM UCB for 48 h, but not 1 µM UCB for 48 h, resulted in a significant increase in the amounts of PPF. These results suggest that the presynaptic function at the Schaffer collateral-CA1 synapses is altered by prolonged UCB exposure only at higher concentrations.

### Prolonged UCB exposure impairs the induction of long-term potentiation and long-term depression

**Figure 2:**
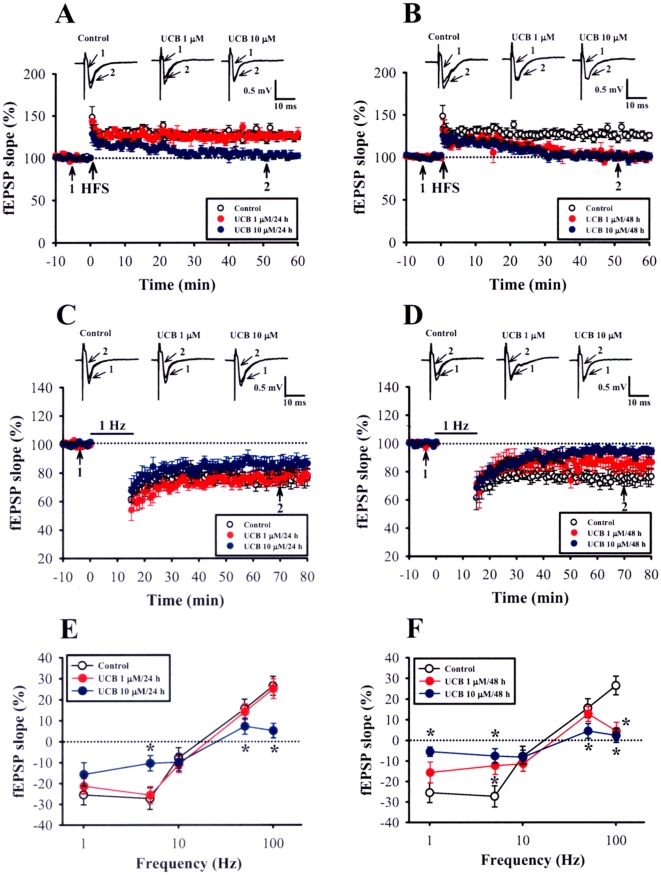
Prolonged UCB exposure impairs the induction of long-term potentiation (LTP) and long-term depression (LTD) in the CA1 region of the hippocampus. (A, B) Summary of experiments showing that the slices from 1 µM UCB exposure for 48 h (B) or 10 µM UCB for 24 h (A) or 48 h (B) displayed a deficit in high frequency stimulation (HFS)-induced (two 1-sec trains of 100 Hz stimuli separated by an intertrain interval of 20 sec) LTP. (C, D) Summary of experiments showing that the slices obtained from control, 1 or 10 µM UCB exposure for 24 h showed a reliable LTD after a prolonged low-frequency stimulation (LFS, 900 stimuli delivered at 1 Hz) (C), whereas slices from 1 or 10 µM UCB exposure for 24 h did not (D). (E, F) Summary of experiments showing the frequency-response curves in slices from control, 1, or 10 µM UCB exposure for 24 h (E) or 48 h (F). The percentage changes in synaptic strength from baseline in all slices were measured at 50 min after stimulation at the indicated frequencies. Error bars indicate SEM. *p<0.05 as compared with the control group by unpaired Student's *t*-test.

To examine the effects of prolonged UCB exposure on long-term synaptic plasticity, we analyzed the induction of LTP and LTD in the CA1 region of the hippocampus. We first examined LTP induced by two 1-sec trains of 100 Hz stimuli separated by intertrain interval of 20 sec, a protocol that normally produces a stable LTP of fEPSPs. In control slices, this protocol consistently induced a robust LTP of fEPSPs, whereas in slices treated with 1 µM UCB for 48 h or 10 µM UCB for 24 h or 48 h, LTP was significantly impaired (50 min after HFS: control, 126.5±5.2% of baseline, n = 21; 1 µM UCB for 48 h, 102.5±4.5% of baseline, n = 11; 10 µM UCB for 24 h, 105.3±5.5% of baseline, n = 8; 10 µM UCB for 48 h, 98.6±3.4% of baseline, n = 10; *p*<0.05) (Figure 2A and B). However, no change in LTP induction was observed in slices treated with 1 µM UCB for 24 h (123.4±3.9% of baseline, n = 8; *p*>0.05) (Figure 2A). A LTD-inducing low-frequency stimulation (LFS, 1 Hz lasting 15 min) was then delivered to the Schaffer collateral afferent fibers. As shown in Figure 2C, following the LFS, there was a robust LTD of fEPSPs in control slices (50 min after the end of LFS: 76.5±4.3% of baseline, n = 16). The magnitude of LTD was not significantly affected by treatment of the slices with 1 µM UCB for 24 h (81.6±4.8% of baseline, n = 8) or 48 h (84.2±5.1% of baseline, n = 8) or 10 µM UCB for 24 h (84.5±4.5% of baseline, n = 8). In contrast, LTD was not induced by LFS in slices treated with 10 µM UCB for 48 h (93.5±3.2% of baseline, n = 7) (Figure 2D). To confirm that the observed deficits in LTP and LTD are not due to the residual UCB in the slices, we tested the effect of acute application of UCB (10 µM) on the induction of LTP and LTD in age-matched slice cultures at 5 days *in vitro* (DIV). Compared with *the* control slices, bath application of 10 µM UCB had no significant effect on basal synaptic responses and the induction of LTP (132.6±5.3% of baseline, n = 5) and LTD (79.8±3.8% of baseline, n = 5) (Figure S2). These results rule out a possible role of residual UCB in the slices in governing the deficits of LTP and LTD occurred after prolonged UCB exposure.

The observations that prolonged UCB exposure impairs LTP and LTD induction suggested the possibility that it may produce an alteration of synaptic modification properties. To explore this possibility, we stimulated the Schaffer collateral afferent fibers with a range of frequencies (5, 10, and 50 Hz) and examined the consequent changes in the synaptic strength. Our results experimentally confirmed the theoretical model of synaptic plasticity originally postulated by Bienenstock, Cooper, and Munro (BCM) [23]; HFS leads to LTP, intermediate frequency stimulation produces only a minor or no change in synaptic strength, and LFS produces LTD. In control slices, 900 pulses of 1 Hz and 5 Hz stimulation induced a LTD of synaptic strength. Moreover, two 1-sec trains of 50 Hz or 100 Hz stimuli induced a significant LTP of synaptic strength. No change in the frequency-response curve was observed in slices treated with 1 µM UCB for 24 h (Figure 2E). In contrast, both the induction of LTD by 5 Hz LFS and the induction of LTP by 50 Hz or 100 Hz by HFS were impaired in slices treated with 1 µM UCB for 48 h or 10 µM UCB for 24 and 48 h, respectively (Figure 2E and F). Together these results suggest that prolonged UCB exposure can induce a time- and concentration-dependent impairment of the inducibility for synaptic modification at the Schaffer collateral-CA1 synapses.

**Figure 3:**
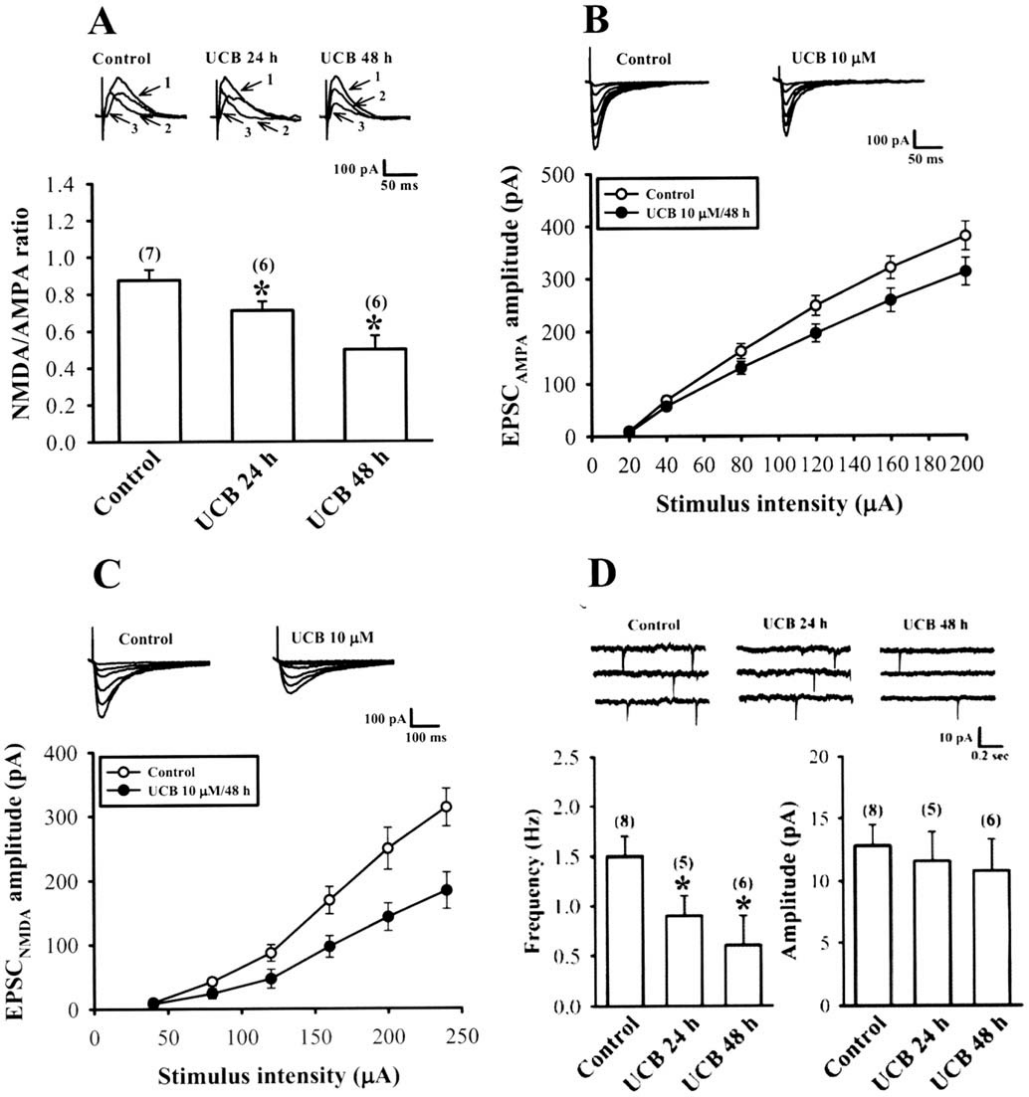
Effect of prolonged UCB exposure on NMDA/AMPA ratio of excitatory postsynaptic currents (EPSCs) and AMPA receptor-mediated miniature EPSCs (mEPSCs). (A) NMDA/AMPA ratio of EPSC (at +50 mV) was determined by subtracting the averaged traces obtained in 50 µM D-APV from those collected in its absence and was found to be significantly lower in slices treated with 10 µM UCB for 24 or 48 h compared with control slices. The intensity of each stimulation was adjusted to evoke the same peak amplitude of EPSCs (≈250 pA) in each slice culture. Representative traces show EPSCs before (1) and after application of D-APV (50 µM) (2) in control and 10 µM UCB-treated slices. The NMDA receptor-mediated component (3) was derived by subtracting the AMPA receptor-mediated component (2) from the compound EPSC (1). (B) Representative traces and input-output curves of AMPA receptor-mediated EPSC (EPSC_AMPA_; at −70 mV in the presence of 20 µM bicuculline methiodide and 50 µM D-APV) versus stimulus intensity (µA) at the Schaffer collateral-CA1 synapses of hippocampal slice cultures in the absence (control) or presence of 10 µM UCB for 48 h. (C) Representative traces and input-output curves of NMDA receptor-mediated EPSC (EPSC_NMDA_; at −60 mV in the presence of 20 µM bicuculline methiodide and 20 µM CNQX) versus stimulus intensity (µA) at the Schaffer collateral-CA1 synapses of hippocampal slice cultures in the absence (control) or presence of 10 µM UCB for 48 h. (D) Representative voltage-clamp recordings of AMPA receptor-mediated mEPSCs (at −70 mV in the presence of 20 µM bicuculline methiodide, 50 µM D-APV, and 1 µM tetrodotoxin) from control slices (left) or slices treated with 10 µM UCB for 24 h (middle) or 48 h (right). The bar graphs show mean±SEM of the effects of UCB on the average frequency and amplitude of mEPSCs. Number of experiments is indicated in the *parenthesis*. *p<0.05 as compared with the control group by one-way ANOVA (Tukey-Kramer test).

Having established that prolonged UCB exposure impairs the induction of both LTP and LTD, we next asked whether these effects are mediated by an alteration of NMDA receptor function, which is critical determinant for the induction of LTP and LTD in the hippocampal CA1 region [16], [18]. To address this issue, we compared the ratio of NMDA to AMPA receptor components of evoked excitatory postsynaptic currents (EPSCs) in CA1 pyramidal neurons of control and UCB-treated slices. We recorded EPSCs when the cell was clamped at +50 mV. In this condition, both NMDA and AMPA receptors are activated by synaptically released glutamate, and their respective contribution to the EPSCs was determined by pharmacological application of NMDA receptor antagonist D-APV (50 µM). Exposure of slices to 10 µM UCB for 24 h (0.71±0.04, n = 6; *p*<0.05) or 48 h (0.49±0.08, n = 6; *p*<0.05) underwent a significant reduction in the NMDA/AMPA ratio when compared with control slices (0.87±0.05, n = 7) (Figure 3A).

A reduction in the NMDA/AMPA ratio could reflect a reduction in the function of NMDA receptors, an increase in the function of AMPA receptors, or a combination of both. To distinguish between these possibilities, we first examined the effect of UCB on pharmacologically isolated AMPA receptor-mediated EPSC (EPSC_AMPA_) recorded at a holding potential of −70 mV in the presence of GABA_A_ receptor antagonist bicuculline methiodide (20 µM) and NMDA receptor antagonist D-APV (50 µM). As shown in Figure 3B, the stimulus-response curve of EPSC_AMPA_ was shifted to the right for the slices exposure of 10 µM UCB for 48 h compared with curve generated from the control slices, confirming a UCB-related reduction in synaptic strength. In addition, the NMDA receptor-mediated EPSC (EPSC_NMDA_) was also isolated at a holding potential of −60 mV in Mg^2+^-free aCSF solution containing bicuculline methiodide (20 µM) and CNQX (20 µM). Similarly, the stimulus-response for EPSC_NMDA_ was decreased in slices treated with 10 µM UCB for 48 h compared with control slices (Figure 3C). Interestingly, at a concentration of 10 µM treatment for 48 h, UCB caused a significantly greater reduction of the amplitude of EPSC_NMDA_ (by ≈45%) than that of EPSC_AMPA_ (by ≈25%). Theoretically, equal depression of the AMPA and NMDA components would indicate involvement of exclusively presynaptic mechanisms, whereas differences in depression would also indicate involvement of postsynaptic mechanisms. These results therefore suggest that prolonged UCB exposure not only causes a decrease in presynaptic transmitter release but also impairs the postsynaptic NMDA receptor function.

To further verify the effect of UCB on presynaptic transmitter release, we recorded AMPA receptor-mediated miniature EPSCs (mEPSCs). As shown in Figure 3D, in slices treated with 10 µM UCB for 24 h (0.9±0.2 Hz, n = 5; *p*<0.05) or 48 h (0.6±0.3 Hz, n = 6; *p*<0.05), the mean frequency of mEPSCs was significantly reduced compared with the control slices (1.5±0.2 Hz, n = 8). No change in the mean amplitude of mEPSCs was observed in slices treated with 10 µM UCB for 24 or 48 h. Together, these results suggest that the decrease in the NMDA/AMPA ratio of EPSCs is mainly attributed to a greater inhibition of UCB on NMDA receptor-mediated postsynaptic response.

We also compared inhibitory postsynaptic currents (IPSCs) recorded in CA1 pyramidal neurons of control and UCB-treated slices. Monosynaptic IPSCs were evoked while clamping the cell at −70 mV in the presence of CNQX (20 µM) and D-APV (50 µM). Figure S3 depicts the relationship between stimulus intensities and IPSC amplitudes. We found that the stimulus-response relationships in slices from control and 10 µM UCB treatment for 48 h are essentially identical, indicating that there are no obvious changes in GABA-mediated synaptic transmission after prolonged UCB exposure.

### Prolonged UCB exposure increases proteolytic cleavage of NMDA receptor subunits by calpain

**Figure 4:**
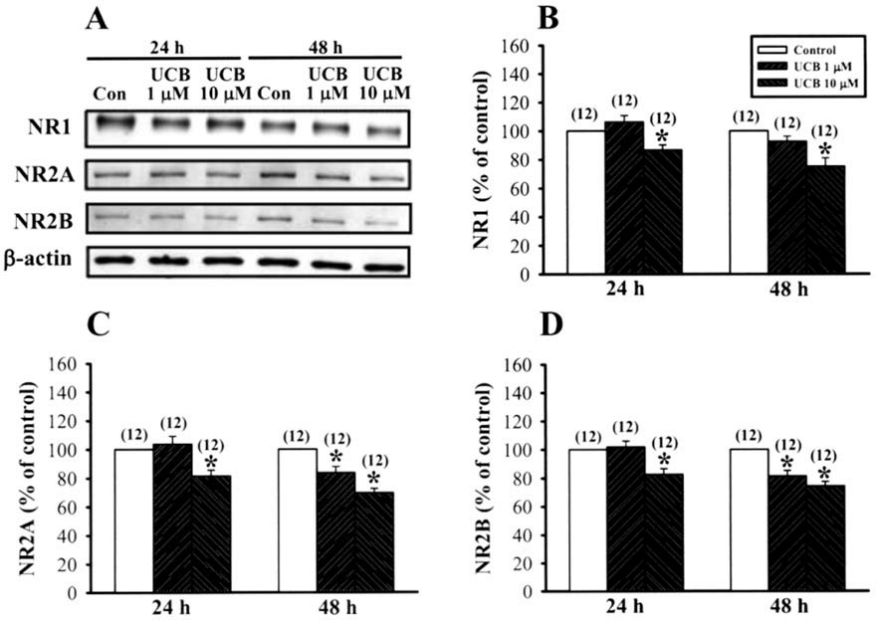
Prolonged UCB exposure decreases the protein levels of NMDA NR1, NR2A and NR2B subunits in rat organotypic slice cultures. (A) Representative immunoblots showing 10 µM UCB treatment for 48 h decreases NR1, NR2A and NR2B subunit expression in the hippocampal CA1 homogenate fractions. (B–D) Corresponding densitometric analysis showing the relative levels of NR1 (B), NR2A (C), and NR2B (D) subunits similar to those shown in (A). Number of experiments is indicated in the *parenthesis*. Error bars indicate SEM. *p<0.05 as compared with the control group by unpaired Student's *t*-test.

We next identified the possible mechanisms underlying the reduction of NMDA receptor-mediated synaptic transmission by prolonged UCB exposure. A decrease in the NMDA receptor-mediated synaptic transmission could reflect a decrease in the number of NMDA receptors. To test this possibility, we compared the protein expression levels of NMDA receptor subunits in the CA1 region of control and UCB- treated slices by Western blot analysis. Using antibodies that selectively label NR1, NR2A, or NR2B subunits, we found that the protein levels of NR1, NR2A, and NR2B subunits were significantly decreased by 10 µM UCB treatment for 24 or 48 h (Figure 4). However, no change in the levels of NR1, NR2A, and NR2B subunits was observed in slices treated with 1 µM UCB for 24 h. Level of β-actin was not altered significantly by UCB.

**Figure 5:**
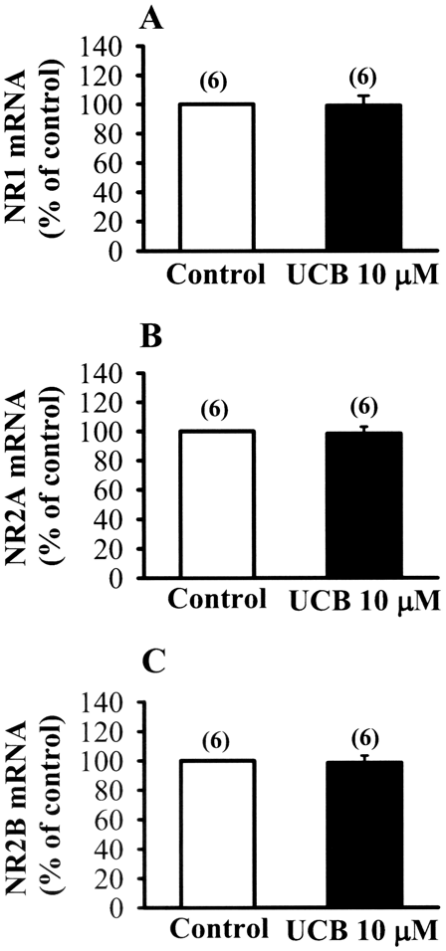
Effect of prolonged UCB exposure on the expression of NR1, NR2A and NR2B mRNAs. (A–C) Real time-PCR analysis showing the relative expression of hippocampal CA1 NR1 (A), NR2A (B) and NR2B (C) mRNAs in slices from control and 10 µM UCB treatment for 48 h. Number of experiments is indicated in the parenthesis. Error bars indicate SEM.

The decrease in the protein expression levels of NMDA receptor subunits could be preceded by a decrease in gene expression. Real-time polymerase chain reaction analysis showed that the mRNA expression profiles for NR1, NR2A, and NR2B subunits were not significantly changed by 10 µM UCB treatment for 48 h (*p*<0.05) (Figure 5). Thus, UCB-induced decrease in NMDA receptor subunit protein expression is not due to the change of gene expression profiles.

**Figure 6:**
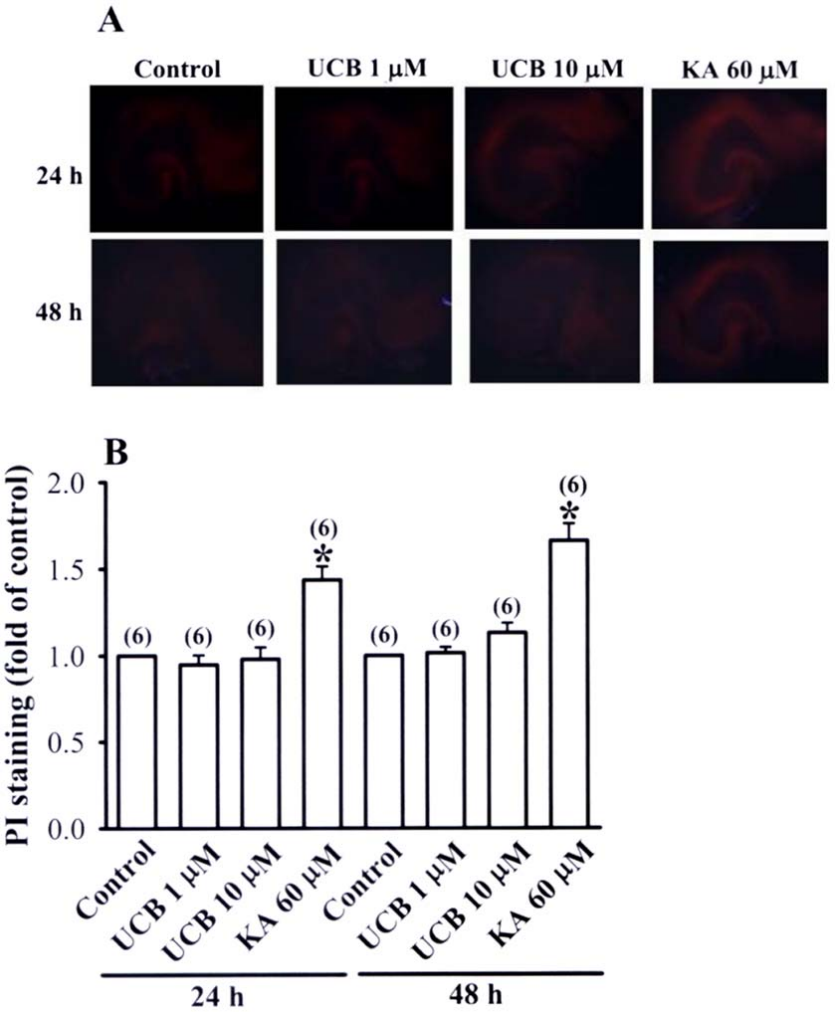
Effects of prolonged UCB exposure on propidium iodide (PI) staining in hippocampal slice cultures. (A) Representative images of PI staining in slices from control or treatment with UCB (1 µM or 10 µM) or kainic acid (60 µM) for 24 or 48 h. (B) Densitometry quantification of PI staining similar to those shown in (A). Data are expressed as fold of increase over the respective control group. Number of experiments is indicated in the *parenthesis*.

It has been shown that UCB can reduce glutamate uptake in astrocytes and in turn induces neuronal injury or death [24], [25]. We therefore asked whether the impairment of LTP and LTD induction observed in UCB-treated slices is attributed to UCB-mediated excitotoxicity. With propidium iodide (PI) staining, UCB (1 or 10 µM) treatment for 24 h or 48 h did not result in higher PI uptake than in control slices (Figure 6A and B). In contrast, exposure of slice to kainic acid (60 µM) for 24 or 48 h resulted in a significant increase in the PI staining. These results indicate that the UCB treatment regimen used in the present study did not affect neuronal viability.

**Figure 7:**
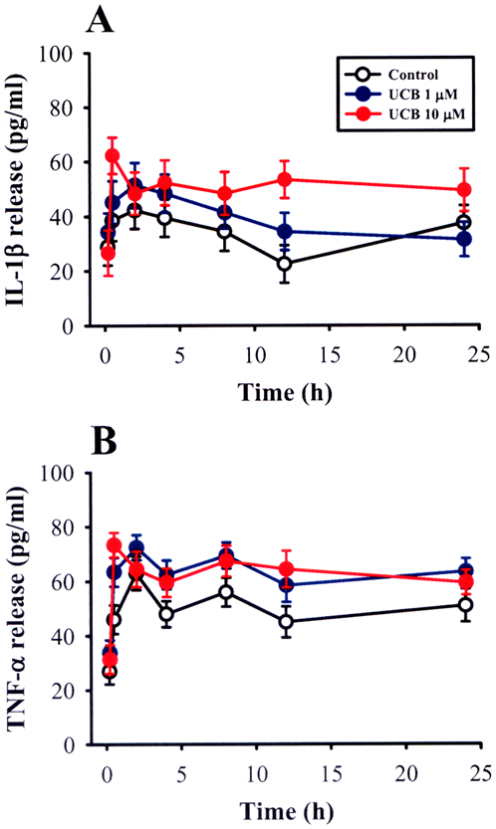
Effects of prolonged UCB exposure on IL-1β and TNF-α release in hippocampal slice cultures. Hippocampal slice cultures were treated with 1 or 10 µM for the indicated time period. IL-1β (A) and TNF-α (B) concentrations in medium were determined by ELISA. Error bars indicate SEM. A two-way repeated measure ANOVA was used to compare the differences of the levels of IL-1β and TNF-α release between groups and changes in post UCB treatment measurements over time. Data are obtained from 5–8 independent experiments performed in duplicate.

Previous studies have shown that prolonged exposure of astrocytes to UCB can trigger release of pro-inflammatory cytokine release such as tumor necrosis factor (TNF)-α and interleukin (IL)-1β [26], [27]. Because these pro-inflammatory cytokines have an inhibitory effect on the induction of both LTP and LTD in the hippocampal CA1 region [28]–[30], we next investigated whether such mechanism could provide a possible explanation for the impairment of LTP and LTD induction observed in UCB-treated slices. A two-way repeated measure ANOVA of IL-1β showed no significant interaction among group and time course (*F*_6,116_ = 1.52, *p* = 0.14) (Figure 7A). Likewise, no significant increase in the level of TNF-α release was observed in slices treated with 1 µM or 10 µM UCG for 24 h compared with the control slices (group×time interaction: *F*_6,137_ = 0.64, *p* = 0.29) (Figure 7B). These results exclude the involvement of IL-1β or TNF-α in the inhibitory effect of UCB on hippocampal CA1 LTP and LTD.

**Figure 8:**
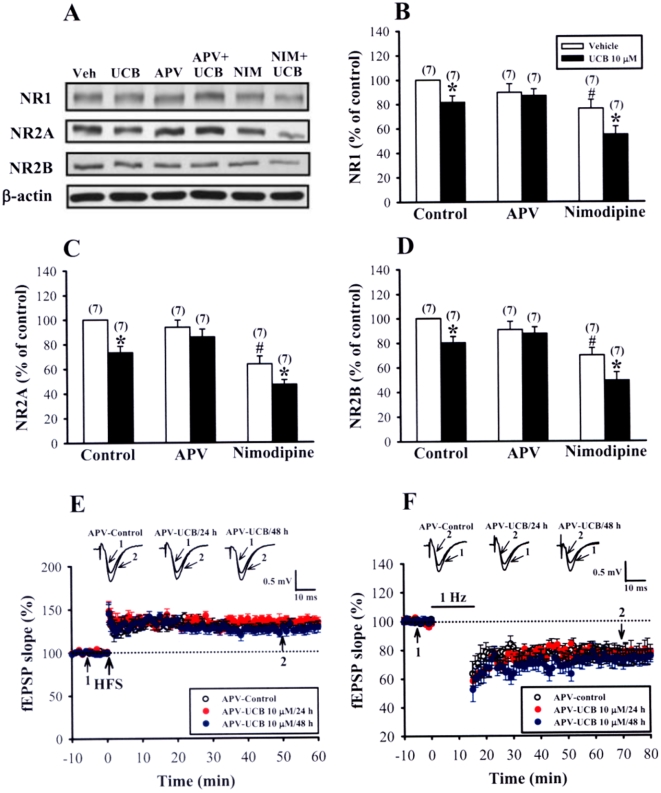
Prevention of UCB-mediated inhibitory effects by blockade of NMDA receptors in hippocampal slice cultures. (A) Representative immunoblots showing protein levels of NR1, NR2A, and NR2B subunits in slices treated with 10 µM UCB for 48 h in the absence or presence of NMDA receptor antagonist D-APV (50 µM) or L-type voltage-gated Ca^2+^ channel blocker nimodipine (10 µM). (B–D) Corresponding densitometric analysis showing the relative levels of NR1 (B), NR2A (C), and NR2B (D) subunits similar to those shown in (A). Number of experiments is indicated in the *parenthesis*. *p<0.05 as compared with the UCB alone group by unpaired Student's *t*-test. #*p*<0.05 as compared with the control group by unpaired Student's *t*-test. (E) Summary of experiments showing the induction of HFS-induced LTP in slices simultaneously treated with 10 µM UCB with D-APV (50 µM) for 24 or 48 h. (F) Summary of experiments showing the induction of LFS-induced LTD in slices simultaneously treated with 10 µM UCB with D-APV (50 µM) for 24 or 48 h. The superimposed fEPSPs in the inset illustrates respective recordings from example experiments taken at the time indicated by number. Error bars indicate SEM.

Since a number of previous studies have proposed a role of NMDA receptors in UCB-induced brain injury [31]–[33], we then examined whether UCB would exert its inhibitory effect on hippocampal synaptic plasticity through NMDA receptor-mediated mechanisms. Simultaneous treatment of slices with UCB (10 µM) and the NMDA receptor antagonist D-APV (50 µM) for 48 h induced no significant changes in the levels of NR1, NR2A, and NR2B subunits (Figure 8A–D). We also evaluated the contribution of the L-type voltage-gated Ca^2+^ channels in the UCB-induced down-regulation of NMDA receptor subunit proteins. However, blockade of L-type voltage-gated Ca^2+^ channels by nimodipine (10 µM) did not prevent a decrease in the expression of NMDA receptor subunits induced by 10 µM UCB treatment for 48 h. In parallel experiments, we also found that D-APV almost completely abolished UCB-induced impairment of LTP (APV-control: 132.5±5.3% of baseline, n = 10; APV-UCB 10 µM for 24 h: 134.5±5.7% of baseline, n = 7; APV-UCB 10 µM for 48 h: 129.7±5.2% of baseline, n = 7; *p*>0.05) and LTD induction (APV-control: 76.5±4.4% of baseline, n = 16; APV-UCB 10 µM for 24 h: 79.4±5.3% of baseline, n = 9; APV-UCB 10 µM for 48 h: 74.7±5.1% of baseline, n = 7; *p*>0.05) (Figure 8E and F). In contrast, UCB-induced decrease in the frequency of mEPSCs was not significantly affected by co-treatment with D-APV (UCB: 0.6±0.3 Hz, n = 6; APV-UCB: 0.8±0.3 Hz, n = 5; *p*>0.05) (Figure S4). In this series of experiments, to prevent the residual D-APV in the slices to affect the induction of LTP and LTD, slices were maintained in the perfusing chamber for a minimum of 1 h prior to recording, a period at least over which D-APV was washed out of the slices. These results indicate a decisive role of NMDA receptors in mediating the cellular effects of UCB.

**Figure 9:**
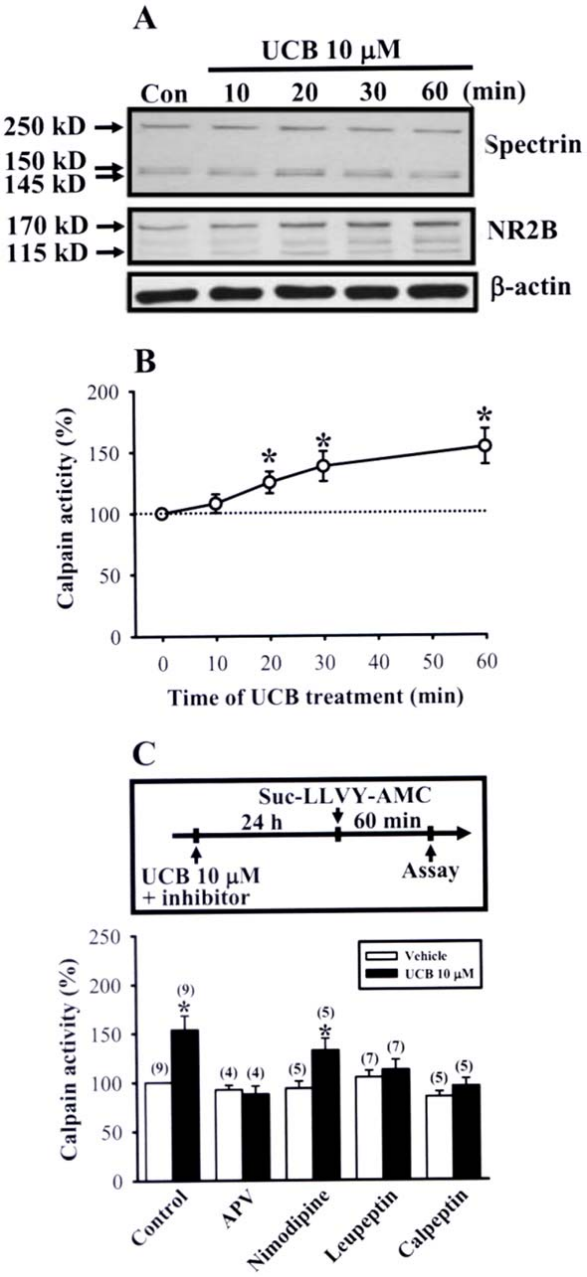
Calpain activation in response to UCB treatment in hippocampal slice cultures. (A) Representative immunoblots showing UCB (10 µM)-induced a time-dependent cleavages of α-spectrin and NR2B subunit protein for the indicated time period. (B) Summary of experiments showing UCB (10 µM)-induced a time-dependent increase in calpain activity. Calpain activity was measured by cleavage of the fluorescent substrate Suc-LLVY-AMC (80 µM). (C) Schematic representation of the protocols used for studying the basal calpain activity after 10 µM UCB treatment for 24 h. The bar graphs show mean±SEM of the effects of 10 µM UCB treatment for 24 h on the basal calpain activity in the absence or presence D-APV (50 µM), nimodipine (20 µM), leupeptin (100 µM), and calpeptin (100 µM). *p<0.05 as compared with the vehicle control group by unpaired Student's *t*-test.

On the basis of the data demonstrating the downregulation of NR1, NR2A and NR2B subunit levels after prolonged UCB exposure, it was assumed that the decrease in the number of NMDA receptors may account for the inhibitory effects of UCB on LTP and LTD induction. One mechanism that could produce this effect is an increase in the proteolytic cleavage of NMDA receptor subunits. Because biochemical studies have clearly demonstrated that the c-terminal region of the NR2 subunit is a substrate for the calcium-activated neutral protease calpain [34]–[36], we sought to assess whether calpain-mediated NR2 subunit cleavage is involved in the mechanism leading to UCB-induced decrease in NMDA receptor number. To test this premise, we first used calpain-mediated spectrin and NR2B subunit cleavage as markers for calpain activation. Western blot analysis found that UCB (10 µM) treatment significantly induced a time-dependent increase in spectrin and NR2B subunit cleavage compared with control slices (Figure 9A). The level of β-actin, a poor calpain substrate [37], was not affected. To directly quantify total amount of intracellular calpain activity, a fluorescent peptide Suc-LLVY-AMC was used as a substrate for calpain [38]. Similarly, we observed a significant increase in calpain activity within 20 min after UCB (10 µM) treatment and the levels were stable over time (Figure 9B). The increased levels of calpain activity correlated temporally with the spectrin and NR2B subunit cleavage analyzed by Western blot analysis. Furthermore, in slices treated with 10 µM UCB for 24 h, the calpain activity was significantly increased (153.6±14.5%, n = 9; *p*<0.05), which was completely blocked by simultaneous addition of D-APV (50 µM, 87.9±8.2%, n = 4) or the specific calpain inhibitors, leupeptin (100 µM, 112.1±10.2%, n = 7) and calpeptin (100 µM, 95.3±7.6%, n = 5) (Figure 9C).

**Figure 10:**
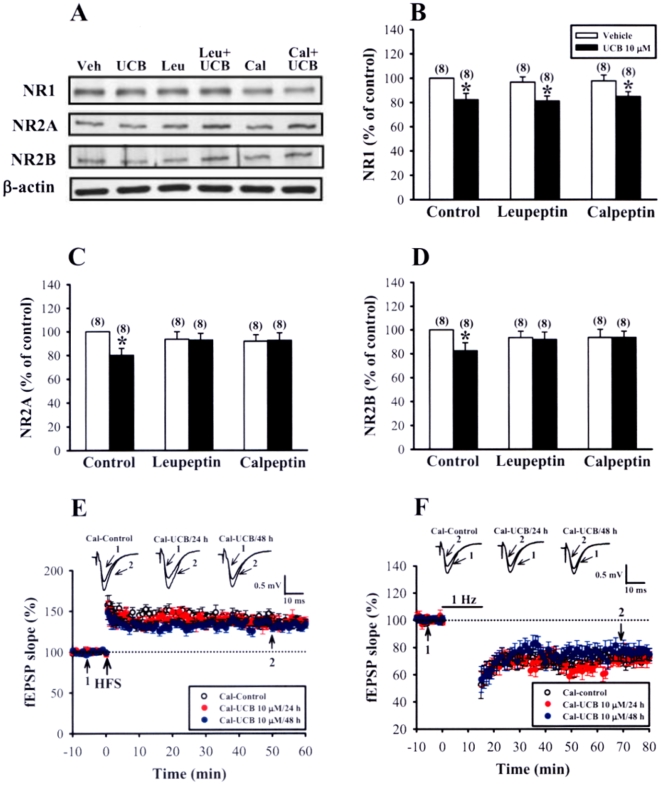
Effects of calpain inhibitors on UCB-induced inhibitory effects in hippocampal slice cultures. (A) Representative immunoblots showing protein levels of NR1, NR2A, and NR2B subunits in slices treated with 10 µM UCB for 48 h in the absence or presence of calpain inhibitors, leupeptin (100 µM) or calpeptin (100 µM). (B–D) Corresponding densitometric analysis showing the relative levels of NR1 (B), NR2A (C), and NR2B (D) subunits similar to those shown in (A). Number of experiments is indicated in the *parenthesis*. *p<0.05 as compared with the UCB alone group by unpaired Student's *t*-test. (E) Summary of experiments showing the induction of HFS-induced LTP in slices simultaneously treated with 10 µM UCB with calpeptin (100 µM) for 24 or 48 h. (F) Summary of experiments showing the induction of LFS-induced LTD in slices simultaneously treated with 10 µM UCB with calpeptin (100 µM) for 24 or 48 h. The superimposed fEPSPs in the inset illustrates respective recordings from example experiments taken at the time indicated by number. Error bars indicate SEM.

To examine the functional consequences of the blockade of calpain activity on the UCB effects, slices were co-exposure to UCB (10 µM) and leupeptin or calpeptin. As shown in Figure 10A–D, both leupeptin (100 µM) and calpeptin (100 µM) completely prevented UCB-induced down-regulation of NR2A and NR2B subunit proteins. However, UCB-induced decrease in NR1 subunit was not affected by addition of either leupeptin or calpeptin (Figure 10B). In parallel experiments, we also found that calpeptin completely abolished UCB-induced impairment of LTP (calpeptin-control: 142.5±5.6% of baseline, n = 10; calpeptin-UCB 10 µM for 24 h: 135.2±6.7% of baseline, n = 8; calpeptin-UCB 10 µM for 48 h: 129.5±4.5% of baseline, n = 8; *p*>0.05) and LTD induction (calpeptin-control: 72.6±2.3% of baseline, n = 10; calpeptin-UCB 10 µM for 24 h: 69.5±4.5% of baseline, n = 9; calpeptin-UCB 10 µM for 48 h: 75.6±5.4% of baseline, n = 7; *p*>0.05) (Figure 10E and F). However, simultaneous treatment with calpeptin had no significant effect on UCB-induced decrease in mEPSC frequency (UCB: 0.6±0.3 Hz, n = 6; calpeptin-UCB: 0.7±0.2 Hz, n = 4; *p*>0.05) (Figure S4). Together, these results suggest that calpain activation mediates UCB-induced down-regulation NMDA receptor number and the impairment of LTP and LTD induction in the hippocampal CA1 region.

## Discussion

Although the toxic effects of UCB have been documented in numerous biological systems, the molecular mechanisms underlying its neurotoxicity have not yet been fully clarified. The present study demonstrates that prolonged exposure of clinically relevant concentrations of UCB impairs the induction of hippocampal CA1 LTP and LTD in rat organotypic slice cultures. These UCB-mediated inhibitory effects are mediated, at least in part, through overstimulation of NMDA receptors, which results in Ca^2+^-induced calpain activation leading to proteolytic cleavage and degradation of NMDA receptor subunits. In addition, we showed that such impairments occur before significant UCB-induced release of the pro-inflammatory cytokines TNF-α and IL-1β or a decrease in neuronal viability.

It is well established that the induction of LTP and LTD at the Schaffer collateral-CA1 synapses of the hippocampus requires the activation of NMDA receptors [16], [18]. Inhibition of NMDA receptors blocks LTP and LTD induction, although recent studies using pharmacological NMDA receptor subtype blockade approach has proposed that LTP induction is specifically dependent on activation of NR2A-containing receptors and LTD requires activation of NR2B-containg receptors [39], [40]. It is therefore reasonable to hypothesize that a pathological change in the function of NMDA receptors might be involved in UCB-mediated impairment of LTP and LTD. Consistent with this hypothesis, we provide the first evidence that prolonged UCB exposure leads to decreased protein levels of NR1, NR2A, and NR2B subunits. Our results further showed that the NMDA to AMPA ratio of EPSCs is reduced in UCB-treated slices, while no change in the amplitude of AMPA receptor-mediated mEPSCs was observed. Given that the mRNA profiles for NR1, NR2A, and NR2B subunits were not significantly altered following prolonged UCB exposure, it seems likely that UCB-induced decrease in NMDA receptor subunit expression is not due to the change in gene expression mechanisms. Nevertheless, our data do not allow us to rule out the possibility that the abnormal intracellular signaling events downstream of NMDA receptor activation are also involved in the establishment of UCB-induced impairment of hippocampal long-term synaptic plasticity. Further studies are needed, however, to definitively clarify this issue.

The stimulus-response relationships for the fEPSPs and EPSC_AMPA_ were shifted to the right for the slices treated with 10 µM UCB for 48 h, suggesting that prolonged UCB exposure may lead to an inhibition of glutamatergic synaptic transmission. Although a decrease in the synaptic transmission could result from a reduction in the function of postsynaptic AMPA receptors, a reduction in presynaptic glutamate release is a more likely interpretation, because UCB-treated slices exhibited an increase in PPF ratio and a decrease in the frequency of AMPA receptor-mediated mEPSCs. In addition, we observed no differences in the amplitude of mEPSCs between UCB-treated and control slices. The present findings are in line with previous studies demonstrating that short-term exposure to UCB *in vitro* [41] or *in vivo* [42] can lead to an impairment of synaptic transmission. Regarding possible mechanisms by which UCB inhibits glutamate release, one possibility may involve the inhibition of synapsin I phosphorylation [43], which plays an important role in neurotransmitter release process [44].

What mechanism might contribute to UCB-mediated decrease in the NMDA receptor subunit proteins? Because the decrease in levels of NMDA receptor subunits observed in UCB-treated slices is correlated with an increase in calpain activity and the blockade of calpain activation almost completely abolished the effects of UCB, we therefore suggest that calpain activation may be involved. To our knowledge, this is the first demonstration that calpain activation participates in the action of UCB to promote NMDA receptor destruction. These observations are compatible with previous findings showing that the C-terminal regions of NR2A and NR2B subunits are substrates for calpain in situ and calpain-mediated proteolytic cleavage of the C-terminus may result in NMDA receptor degradation and reduced activity [34]–[36]. Interestingly, blockade of NMDA receptors but not L-type voltage-gated Ca^2+^ channels also prevented the UCB-mediated decrease in the NMDA receptor subunits, indicating that activation of NMDA receptors is critical for the stimulation of calpain by UCB. This is in line with previous evidence that NMDA receptor antagonists are capable to prevent UCB-induced neurotoxicity, both *in vivo* [31] and *in vitro* experiments [32], [33]. A pressing question that follows from these observations is that how UCB might affect NMDA receptor activation. So far, there is no evidence that UCB can activate NMDA receptors directly. One possible mechanism is that UCB may decrease the uptake of glutamate and thus prolong the presence of glutamate in the synaptic cleft, which ultimately lead to overstimulation of NMDA receptors [45]. An alternative, but not mutually exclusive, mechanism may involve functional interactions between UCB and NMDA receptors leading to the enhanced NMDA receptor function. Previous evidence has shown that that UCB exposure *in vivo* in newborn piglets can increase the binding affinity of NMDA receptors for MK-801 [46].

A recent study has emphasized the importance of pro-inflammatory cytokine TNF-α and IL-1β release in UCB-induced loss of cell viability [27]. In contrast to this view, however, we found no significant changes in the levels of TNF-α and IL-1β after UCB exposure. The reason for this discrepancy is not clear but could be attributed to the use of different doses of UCB challenges (50 µM *versus* 10 µM) or cultured model systems (astroglial cell cultures *versus* organotypic hippocampal slice cultures), resulting in stimulating different cellular processes that may vary in their mode of action. In accordance, we did not find significant changes in cell viability after UCB exposure. One argument might be that the organotypic slice culture model is not suitable for studying inflammatory reactions. Indeed, there is excellent evidence showing that this system is an ideal model to study the functional consequences of changes in inflammatory responses caused by acute or chronic excitotoxic insults [47], [48].

The concentration of UCB in the brain and the duration of exposure to UCB are important determinants of the development of UCB neurotoxicity [49]–[51]. Virtually most published studies of UCB-mediated neurotoxicity have been carried out at concentrations of UCB that exceeded those seen in jaundiced neonates with clinical signs of bilirubin encephalopathy, rendering uncertain relevance for the observations to the clinical manifestations of neurotoxicity [50]. To exclude this concern, we studied the effects of UCB on the induction CA1 LTP and LTD in developing rat organotypic hippocampal slice cultures at theoretically calculated free fraction concentrations of 3 to 30 nM. It is evident that jaundiced neonates have total serum bilirubin concentrations of 7 to 17 mg/dL and serum bilirubin concentrations higher than 20 mg/dL may cause kernicterus [49], [52]. Using the corrected affinity constants at pH 7.4, it was estimated that the concentration of free UCB may exceed 70 nM of its maximum aqueous solubility when total serum concentration exceeds approximately 5 mg/dL [50]. Therefore, the concentrations of UCB used in the present study are clinically relevant. It is also known that UCB has multiple physical states, including monomers, oligomers, metastable aggregates and sedimentable particles [53]. Given the estimated free concentrations of UCB used in the present study are below its maximum aqueous solubility, it is unlikely that the inhibitory effects of UCB observed in our experimental conditions were mediated by precipitation of UCB aggregates. Instead, it seems that UCB executes its action in monomers or other oligomeric form [54].

In conclusion, our work supports the notion that prolonged exposure of clinically relevant UCB concentrations may lead to a time- and concentration-dependent inhibitor effect on the induction of hippocampal CA1 long-term synaptic plasticity in rat organotypic slice cultures and, more importantly identifies specific molecular mechanisms to support this contention. The data presented here will help elucidate how UCB affects the signaling pathways in the hippocampus, possibly shedding light on how elevated levels of UCB in the infant period cause hippocampal dysfunction leading to temporary or permanent impairment of mental function. Because calpain inhibition can prevent the inhibitory effects of UCB on hippocampal neurons, this study point to calpain inhibitor as a new potential therapeutic approach for UCB-induced neurotoxicity in the hippocampus. Finally, our results indicate that the organotypic hippocampal slice cultures have potential as model system to study mechanisms of UCB neurotoxicity that can be useful to develop novel therapeutic strategies.

## Materials and Methods

### Organotypic hippocampal slice cultures

All experimental procedures were carried out according to the guidelines laid down by the Institutional Animal Care and Use Committee of National Cheng Kung University. Organotypic cultures of the hippocampus were prepared and maintained according to the standard interface method described previously [55], [56]. Briefly, hippocampi from 6- to 8-d-old male Sprague-Dawley rats were dissected and cut into 400 µm slices with a McIlwain tissue chopper (Mickle Laboratory Engineering, Gomshall, Surrey, UK). Only slices from the middle third of the hippocampus were used for culturing. Individual slices that displayed an intact dentate gyrus and pyramidal cell layer were transferred to sterile Millicell-CM (Millipore, Bedford, MA) membrane inserts (0.4 µm) in six-well culture plates with 1 ml growth medium. The growth medium composition was 50% minimal essential medium, 25% Hank's balanced salt solution, 25% heat-inactivated horse serum, 25 mM HEPES, pH 7.3, supplemented with glutamax (Invitrogen Molecular Probes, Carlsbad, CA), glucose (6.5 mg/ml), penicillin (100 units/ml; Invitrogen Molecular Probes) and streptomycin (100 µg/ml; Invitrogen Molecular Probes). The cultures were grown in an incubator with 5% CO_2_ and 95% atmospheric air at 35°C and the medium was changed the day after slicing.

### UCB treatment

Slices were maintained in vitro for a minimum of 3 days prior to use, a period at least over which tissues recover from experimental trauma caused by the isolation procedure [48]. Moreover, the use of this time in culture also matches up to brain growth rate in full-term neonate [57]. At the start of experiments, the culture medium was replaced with 1 ml of serum-free neurobasal medium (Invitrogen Molecular Probes) with 1 mM L-glutamine, and B27 supplement (Invitrogen Molecular Probes). UCB was obtained from Sigma (St Louis, MO) and purified according to the method of McDonagh [58]. UCB (1 or 10 µM), in the presence of human serum albumin (HSA, 2 or 20 µM), was added to the medium for 24 or 48 h at 35°C. A 10 mM UCB stock solution in 0.1 M NaOH was prepared immediately before use under light protection to avoid photodegradation; the pH was restored to 7.4 by addition of equal amounts of 0.1 M HCl. Control slices were processed in parallel without addition of UCB. Under the present experimental conditions, the theoretically calculated free fraction of UCB concentrations were 3 and 30 nM, respectively, according to the model proposed by Weisiger et al. [59]. These free UCB concentrations were below the aqueous solubility limit of 70 nM [50]. In order to characterize the cellular mechanisms by which UCB can affect the induction of LTP and LTD, slice cultures were additionally treated, in some experiments, with calpain inhibitors, NMDA receptor antagonist, or L-type voltage-gated Ca^2+^ channel blocker. For this purpose, the calpain inhibitors, leupeptine (100 µM) and calpeptin (100 µM), NMDA receptor antagonist, D-APV (50 µM), as well as L-type voltage-gated Ca^2+^ channel blocker, nimodipine (10 µM), were added to the culture medium, respectively, just before UCB administration and then left for 24 or 48 h. D-APV, nimodipine, leupeptine, and calpeptin were purchased from Tocris Cookson (Bristol, UK). All reagents were added to medium, which was equilibrated at 35°C, 5% CO_2_ before their addition to the slices. Drug concentrations were selected on the basis of previously published studies or our preliminary results.

### Electrophysiological recordings

Slice cultures on membrane inserts were transferred to a submersion-type recording chamber continually perfused with 30–32°C oxygenated (95% O_2_–5% CO_2_) artificial cerebrospinal fluid (aCSF) solution containing (in mM): 117 NaCl, 4.7 KCl, 2.5 CaCl_2_, 1.2 MgCl_2_, 25 NaHCO_3_, 1.2 NaH_2_PO_4_ and 11 glucose. Extracellular and whole-cell patch-clamp recordings were carried out with Axoclamp-2B or Axopatch 200B amplifier (Axon Instruments, Foster City, CA) as described previously [60]. The responses were low pass filtered at 2 kHz, digitally sampled at 5–10 kHz, and analyzed using pCLAMP software (Version 8.0; Axon Instruments). The evoked postsynaptic responses were induced in CA1 stratum radiatum by stimulation (0.02 ms duration) of Schaffer collateral/commissural afferents at 0.033 Hz with a bipolar stainless steel stimulating electrode. Field EPSPs were recorded with a glass pipette filled with 1 M NaCl (2–3 MΩ resistance) and the initial slope was measured. The strength of synaptic transmission was quantified by measuring the slope of fEPSP. PPF was assessed by using a succession of paired pulses separated by intervals of 20, 40, 60, 80, 100, and 200 ms. LTP was induced by high-frequency stimulation, at the test pulse intensity, consisting of two 1-sec trains of stimuli separated by an intertrain interval of 20 sec at 100 Hz. LTD was induced using a standard protocol of 900 stimuli at 1 Hz. The stimulation intensity during low-frequency stimulation application was the same as the test pulse intensity. Whole-cell recording of EPSCs was made from CA1 pyramidal cells, which were identified under a DIC microscope. For EPSC recordings, patch pipettes (3–5 MΩ) filled with the following internal solution were used (in mM): 130 CsMeSO_3_, 8 NaCl, 10 HEPES, 0.5 EGTA, 4 Na_2_ATP, 0.3 Na_3_GTP, 5 QX-314 *N*-(2,6-Dimethyl-phenylcarbamoylmethyl) triethylammonium bromide; which had an osmolarity of 290–295 mOsm and pH of 7.2. The amplitude of evoked EPSCs was measured. To ensure stability of the whole-cell recordings, electrical stimulation was initiated before the cell was patched. We waited for approximately 5 min in the cell-attached configuration before break-in to wash off any residual internal solution spilled from the approaching pipette. For recording the ratio of NMDA to AMPA receptor component of EPSCs, cells were clamped at +50 mV to reduce Mg^2+^ blockade of the NMDA receptors. The intensity of each stimulation was adjusted to evoke the same peak amplitude of EPSCs (≈250 pA) in each slice culture. The NMDA/AMPA ratio was determined by subtracting the averaged traces obtained in D-APV (50 µM) from those collected in its absence. EPSC_AMPA_ was recorded in the presence of D-APV (50 µM) and bicuculline methiodide (20 µM) at a holding potential of −70 mV. When recording EPSC_NMDA_, cells were clamped at −60 mV in Mg^2+^-free aCSF containing CNQX (20 µM) and bicuculline methiodide (20 µM). For recording IPSCs, cells were clamped at −70 mV in the presence of CNQX (20 µM) and D-APV (50 µM). The composition of intracellular solution for recording IPSCs was (mM): 150 CsCl, 10 HEPES, 2 MgCl_2_, 0.5 EGTA, 4 Na_2_ATP, 0.3 Na_3_GTP, 5 QX-314. In the experiments involving recordings of AMPA receptor-mediated mEPSCs, slices were bathed in the presence of bicuculline methiodide (20 µM), D-APV (50 µM), and tetrodotoxin (1 µM). Neurons were held at −70 mV and spontaneous activity was recorded. The software detects events based on amplitudes exceeding a threshold set just above the baseline noise of the recording (−3 pA). All detected events were re-examined and accepted or rejected based on subjective visual examination. Background current noise was estimated from the baseline with no clear event and was subtracted from signals for analysis. The mEPSC frequencies were calculated by dividing the total number of detected events by the total time sampled. Series and input resistances were monitored throughout each experiment. Data were discarded if series resistance changed more than 15% during an experiment.

### Western Blotting

The microdissected slice samples for each experimental condition were transferred into ice-cold lysis buffer (pH 7.4) containing a cocktail of protein phosphatase and proteinase inhibitors (50 mM Tris-HCl, 100 mM NaCl, 15 mM sodium pyrophosphate, 50 mM sodium fluoride, 1 mM sodium orthovanadate, 5 mM EGTA, 5 mM EDTA, 1 mM phenylmethylsulfonyl fluoride, 1 µM microcystin-LR, 1 µM okadaic acid, 0.5% Triton X-100, 2 mM benzamidine, 60 µg/ml aprotinin, and 60 µg/ml leupeptin) to avoid dephosphorylation and degradation of proteins, and ground with a pellet pestle (Kontes glassware, Vineland, NJ). Samples were sonicated and spun down at 15,000×g at 4°C for 10 min. The supernatant was then assayed for total protein concentration using Bio-Rad Bradford Protein Assay Kit (Hercules, CA). Each sample was separated in 7% SDS-PAGE gel. Following the transfer on nitrocellulose membranes, blots were blocked in TBS containing 3% bovine serum albumin and 0.01% Tween 20 for 1 h and then blotted for 2 h at room temperature with antibody that recognize NR1 (1∶1000; Chemicon, Temecula, CA), NR2A (1∶1000; Santa Cruz Biotechnology, Santa Cruz, CA), NR2B (1∶1000; Santa Cruz Biotechnology), NR2B (an N-terminal antibody to 251 amino acid sequence, 1∶500; Zymed Laboratories Inc., San Francisco, CA), spectrin (1∶500; Biomol, Plymouth Meeting, PA) or β-actin (1∶20000; Sigma-Aldrich, St Louis, MO). It was then probed with HRP-conjugated secondary antibody for 1 h and developed using the ECL immunoblotting detection system (Amersham Pharmacia Biotech, Piscatway, NJ). Immunoblots were analyzed by densitometry using Bio-profil BioLight PC software. Only film exposures that were in the linear range of the ECL reaction were used for quantification analysis. Expression of NR1, NR2A and NR2B was evaluated relative to that for β-actin. Background correction values were subtracted from each lane to minimize the variability across membranes.

### Quantitative Real-Time RT-PCR

Total RNA was isolated from frozen hippocampal CA1 region of the slice cultures using TriReagent kit (Molecular Research Center, Cincinnati, OH), treated with RNase-free DNase (RQ1; Promega, Madison, WI) to remove the potential contamination of genomic DNA. Approximately 1 ng of RNA was reverse-transcribed and amplified following the quantitative one-step real-time RT-PCR technique (Titanium One-Step RT-PCR kit, BD Biosciences Clontech, Palo Alto, CA), with both RT and PCR in the same tube. Real-time RT-PCR was performed on the Roche LightCycler instrument (Roche Diagnostics, Indianapolis, IN) using the FastStart DNA Master SYBR Green I kit (Roche Applied Science) as described by the manufacturer. The PCR mixtures were incubated at 95°C for 10 min, and then 35 PCR cycles were conducted (95°C for 10 sec, 55°C for 15 sec and 68°C for 20 sec). The primer combinations were designed by referring to the rat studies by Pickering et al. [61] and Ku et al. [62]: NR1 5′-CTGCAACCCTCACTTTTGAG-3′ (forward) and 5′-TGCAAA AGCCAGCTGCATCT-5′ (reverse); NR2A, 5′-GACGGTCTTGGGATCTTA AC-3′ (forward) and 5′-TGACCATGAAATTGGTGCAGG-3′ (reverse); NR2B 5′-TGC ACAATTACTCCTCGACG-3′ (forward) and 5′-TCCGATTCTTCTTCTGAGCC-3′ (reverse); β-actin, 5′-TTCTACAATGAGCTGCGTGTGGC-3′ (forward) and 5′-CTCATAGCTCTTCTCCAGGGAGGA-3′ (reverse). Real-time RT-PCR reactions on mRNA obtained from control or neonatal isolated rat hippocampal CA1 total RNA samples were performed at the same time. PCR amplifications were repeated in duplicate. A melting curve was created at the end of the PCR cycle to confirm that a single product was amplified. Data were analyzed by the LightCycler quantification software to determine the threshold cycle above the background for each reaction. The relative transcript amount of the gene of interest, which was calculated using standard curves of serial RNA dilutions, was normalized to that of β-actin of the same RNA.

### Assessment of neuronal damage by propidium iodide uptake

Neuronal death in the slice cultures was assessed by measurement of the fluorescent exclusion dye propidium iodide (PI; Invitrogen Molecular Probes) uptake as described in detail elsewhere [47]. PI only enters cells with damaged cell membrane, interacting with DNA to yield a bright red fluorescence. PI (5 µg/ml) was added to the culture medium together with different treatment. After 3 h incubation, PI fluorescence was visualized using a 5× objective with a fluorescent microscope (Olympus BX51, Tokyo, Japan), and images of PI-labeled slices were captured with a digital camera system (Olympus Optical DP70). Except for adjustment to the contrast and brightness levels, no other manipulations were done in any of the images. Images were analyzed quantitatively by densitometry with NIH Image 1.62 analysis software.

### Quantification of IL-1β and TNF-α release by ELISA

The culture supernatants from control and treated slice cultures at appropriate time points was collected, centrifuged at 600×g for 5 min, and stored in aliquots at −70°C. Concentrations of IL-1β and TNF-α were measured with commercial DuoSet ELISA Development kit (R & D Systems, Minneapolis, MN) according to the manufacturer's instructions. IL-1β and TNF-α levels are expressed as pg/ml.

### Calpain activity assay with Suc-LLVY-AMC

Cleavage of the fluorogenic calpain substrate Succinyl-Leu-Leu-Val-Tyr-7-amino-4-methylcoumarin (Suc-LLVY-AMC; Calbiochem, La Jolla, CA) to its fluorescent product AMC was used to measure intracellular calpain activity as described previously [36]. Suc-LLVY-AMC (80 µM) was added to the culture medium together with different treatment. After 1 h incubation, the slice culture samples for each experimental condition were transferred into ice-cold lysis buffer, sonicated, and spun down at 15,000×g at 4°C for 15 min. The supernatant was then assayed for total protein concentration using Bio-Rad Bradford Protein Assay Kit (Hercules, CA) and for calpain activity assay. The fluorescence was assessed using a 96-well fluorescent microplate reader (Gemini Spectra XPS, Molecular Devices, Union City, CA) with a 365 nm excitation and a 460 nm emission. In some experiments, slice cultures were pretreated Suc-LLVY-AMC (80 µM) for 30 min before UCB added and calpain activity was assayed from 0 to 60 min after UCB application.

### Data Analysis

All data are expressed as means±SEM, and unless stated otherwise, the statistic significance was determined using a one-way analysis of variance (ANOVA) for repeated measurements with a post-hoc Tukey-Kramer test or Student's t-test. We used a two-way repeated measure ANOVA to compare the differences of the levels of IL-1β and TNF-α release between groups and changes in post UCB treatment measurements over time. The Fisher's protected LSD post-hoc test was used for pair-wise comparisons after ANOVA. Probability values of p<0.05 were considered to represent significant differences. All data analyses were performed using SPSS commercially available statistical analysis software (SigmaStat Version 2.0; Chicago, IL, USA).

## Supporting Information

Figure S1 Effects of prolonged UCB exposure on the presynaptic fiber volley. Input-output curves of the amplitude of presynaptic fiber volley versus stimulus intensity (µA) at the Schaffer collateral-CA1 synapses of hippocampal slice cultures in the absence (control, n = 5) or presence of 10 µM UCB for 24 (n = 5) or 48 h (n = 5). Representative traces (average of three responses) show example fiber volley recorded in slices from control and UCB-treated slices in the presence of CNQX (20 µM) and D-APV (50 µM).
(7.01 MB TIF)

Figure S2 Effects of acute UCB exposure on the induction of LTP and LTD in the CA1 region of the hippocampus. (A) Summary of experiments showing that bath application of UCB (10 µM, n = 5) had no significant effect on basal synaptic transmission and the induction of HFS-induced LTP in slice cultures at 5 DIV. (B) Summary of experiments showing that bath application of UCB (10 µM, n = 5) had no significant effect on the induction of LTD by LFS in slice cultures at 5 DIV. Representative traces of fEPSPs were taken at the time indicated by number. Error bars indicate SEM.
(5.33 MB TIF)

Figure S3 Effects of prolonged UCB exposure on the inhibitory postsynaptic currents (IPSCs). Input-output curves of the amplitude of IPSCs versus stimulus intensity (µA) at the Schaffer collateral-CA1 synapses of hippocampal slice cultures in the absence (control, n = 5) or presence of 10 µM UCB for 48 h (n = 5). Representative traces show example IPSCs (average of three responses) recorded in slices from control and UCB-treated slices at −70 mV in the presence of CNQX (20 µM) and D-APV (50 µM).
(4.18 MB TIF)

Figure S4 Effects of D-APV and calpeptin on UCB-induced decrease in the frequency of mEPSCs. The bar graphs show mean±SEM of the effects of UCB (10 µM) on the average frequency (A) and the amplitude (B) of AMPA receptor-mediated mEPSCs in slices simultaneously treated with D-APV (50 µM) or calpeptin (100 µM) for 48 h. Number of experiments is indicated in the parenthesis. *p<0.05 as compared with the control group by one-way ANOVA (Tukey-Kramer test).
(4.09 MB TIF)
