# Supplementary figures and images for: Unconjugated Bilirubin Exposure Impairs Hippocampal Long-Term Synaptic Plasticity
Source: PLoS One. 2009 Jun 11;4(6):e5876. doi: 10.1371/journal.pone.0005876 (PMC2690688; doi:10.1371/journal.pone.0005876)

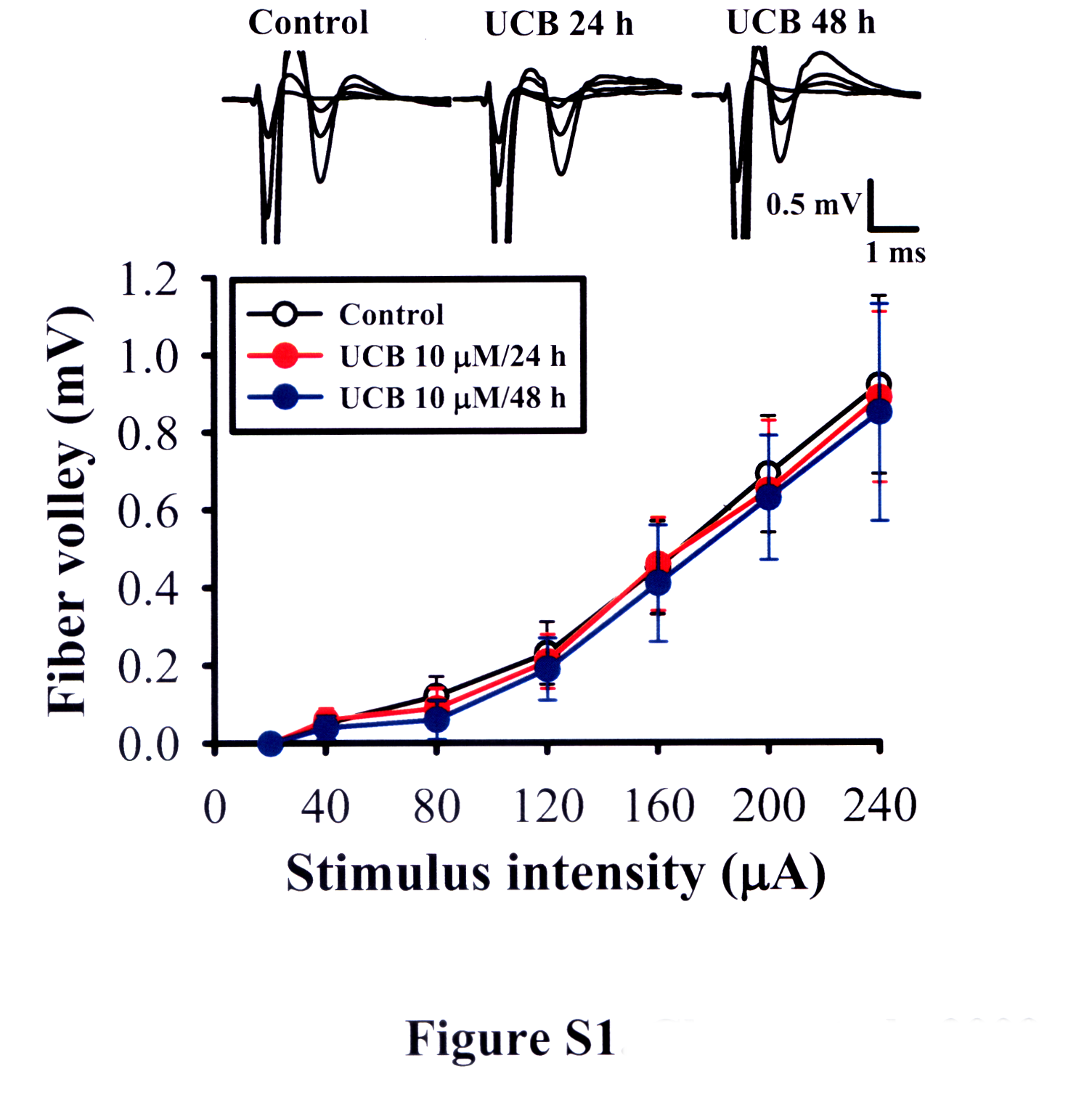

Supplement: Figure S1 — Effects of prolonged UCB exposure on the presynaptic fiber volley. Input-output curves of the amplitude of presynaptic fiber volley versus stimulus intensity (µA) at the Schaffer collateral-CA1 synapses of hippocampal slice cultures in the absence (control, n = 5) or presence of 10 µM UCB for 24 (n = 5) or 48 h (n = 5). Representative traces (average of three responses) show example fiber volley recorded in slices from control and UCB-treated slices in the presence of CNQX (20 µM) and D-APV (50 µM). (7.01 MB TIF) [file pone.0005876.s001.tif]

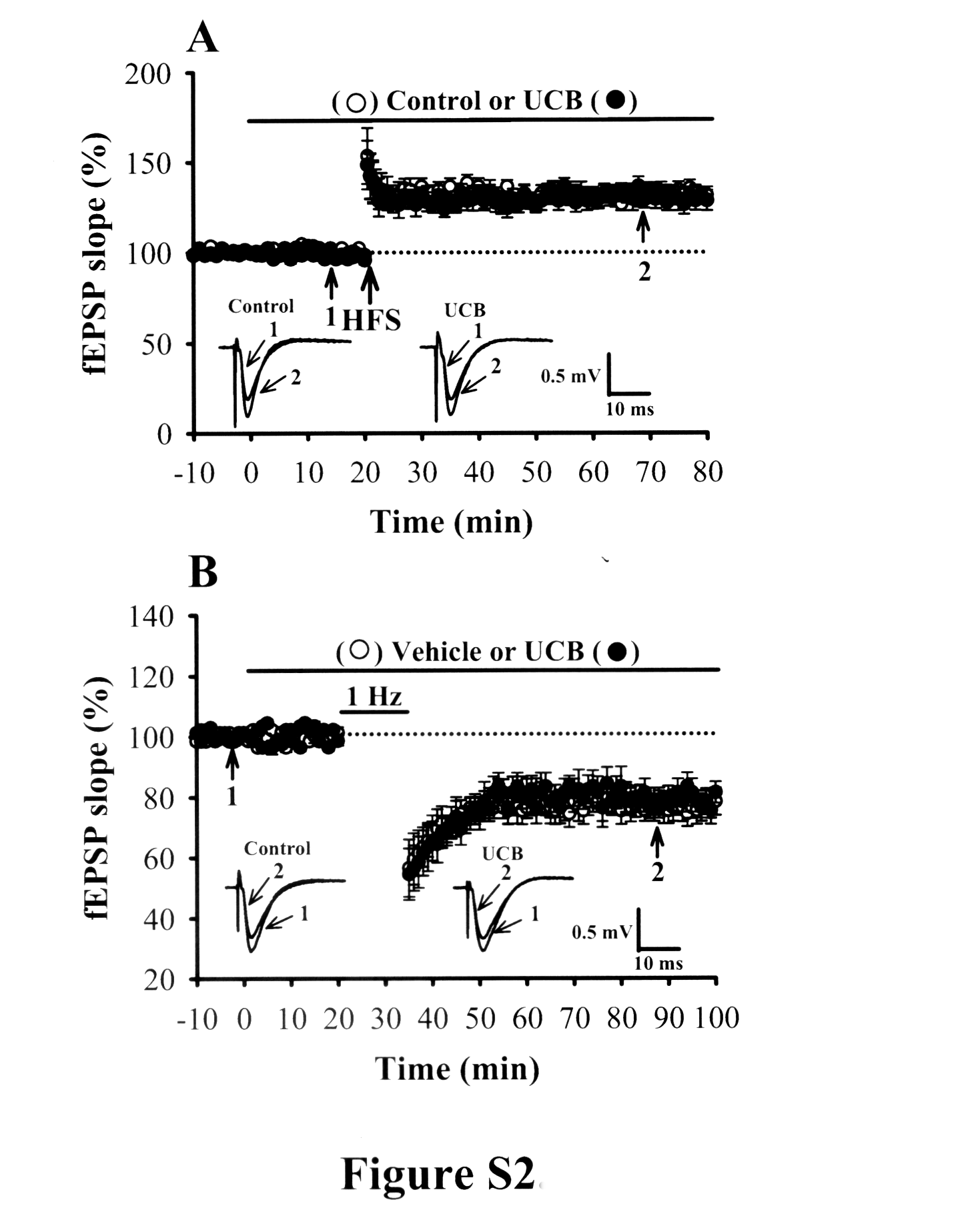

Supplement: Figure S2 — Effects of acute UCB exposure on the induction of LTP and LTD in the CA1 region of the hippocampus. (A) Summary of experiments showing that bath application of UCB (10 µM, n = 5) had no significant effect on basal synaptic transmission and the induction of HFS-induced LTP in slice cultures at 5 DIV. (B) Summary of experiments showing that bath application of UCB (10 µM, n = 5) had no significant effect on the induction of LTD by LFS in slice cultures at 5 DIV. Representative traces of fEPSPs were taken at the time indicated by number. Error bars indicate SEM. (5.33 MB TIF) [file pone.0005876.s002.tif]

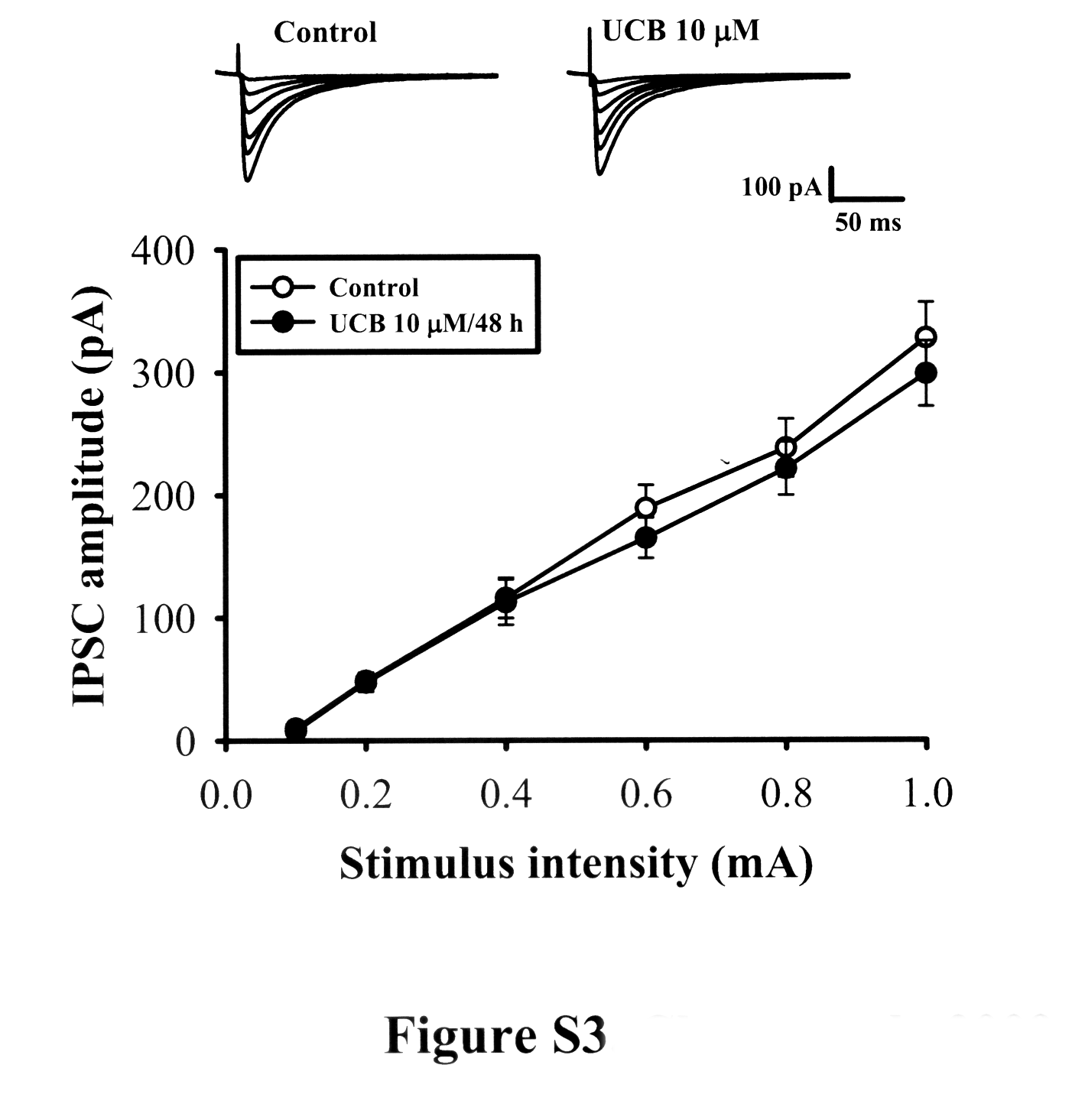

Supplement: Figure S3 — Effects of prolonged UCB exposure on the inhibitory postsynaptic currents (IPSCs). Input-output curves of the amplitude of IPSCs versus stimulus intensity (µA) at the Schaffer collateral-CA1 synapses of hippocampal slice cultures in the absence (control, n = 5) or presence of 10 µM UCB for 48 h (n = 5). Representative traces show example IPSCs (average of three responses) recorded in slices from control and UCB-treated slices at −70 mV in the presence of CNQX (20 µM) and D-APV (50 µM). (4.18 MB TIF) [file pone.0005876.s003.tif]

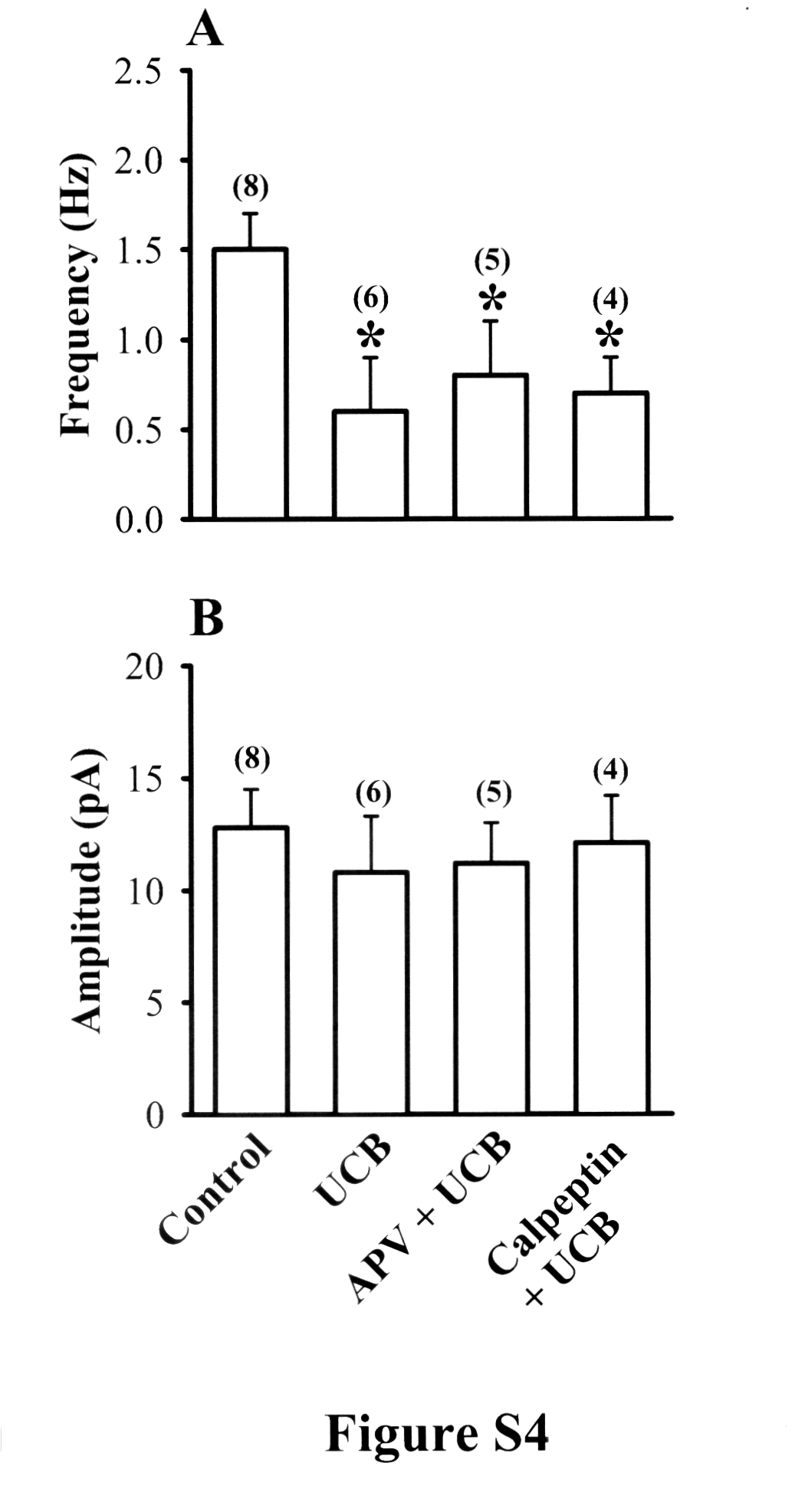

Supplement: Figure S4 — Effects of D-APV and calpeptin on UCB-induced decrease in the frequency of mEPSCs. The bar graphs show mean±SEM of the effects of UCB (10 µM) on the average frequency (A) and the amplitude (B) of AMPA receptor-mediated mEPSCs in slices simultaneously treated with D-APV (50 µM) or calpeptin (100 µM) for 48 h. Number of experiments is indicated in the parenthesis. *p<0.05 as compared with the control group by one-way ANOVA (Tukey-Kramer test). (4.09 MB TIF) [file pone.0005876.s004.tif]
